# Advice as a Form of Structural Coupling: Intersystem Organizations and Scientific Communication in the Japanese Response to COVID-19

**DOI:** 10.1007/s11213-023-09649-9

**Published:** 2023-05-13

**Authors:** Kosuke Sakai

**Affiliations:** Graduate School of Arts and Sciences, The University of Tokyo, 153-8902 3-8-1 Komaba, Meguro-ku, Tokyo, Japan

**Keywords:** Advice, System theory, Scientific communication, Structural coupling

## Abstract

A critical issue in the study of scientific communication from a systems theoretical perspective is its role in multiple intersystem relationships. During COVID-19, politics has adopted scientific findings to inform political decisions. However, science has in response actively coordinated its operations for providing desired stimuli to politics. Luhmann identified advice as a form of structural coupling that links political and scientific systems. Advice is not a monolithic intervention by which one side acts on the other but is rather an interface that enables the two systems to relate through distancing. In this article, I empirically illustrate how the structural coupling of the political system and scientific system through advice manifests itself in an examination of the roles that various organizations (expert meeting and cluster task forces) have played in Japan’s response to COVID-19. Through this analysis, I provide a theoretical insight regarding these organizations and a more detailed case analysis of the transformation of certain organizations to re-describe the system theoretical insights of advice in the form of scientific communication between politics and science.

## Introduction

A central issue in scientific communication concerns the relationship and boundaries between politics and science. Concerning this theme in the social sciences, the focus of theoretical and empirical inquiry has been on situating professional knowledge as a form of advice in a policy making context. The role of advice is unavoidable when considering scientific communication in the post-COVID-19 world. The following questions, for example, must be raised: How are politics and science distinguished in times of crisis? What does this distinction mean in terms of the use of expertise in problem solving? What makes the use of expertise possible?

These questions have been investigated in the field of science and technology studies as bearing on the relationship between science and politics (Kropp and Wagner 2010: 813). However, this analysis has generally focused on the position that science (scientists) can take with respect to politics (politicians) from its, but too little consideration has been granted to how this expertise has been received in the political process (Kropp and Wagner 2010: 834). Therefore, the ways that scientific communication has become politicized in course of policy making remains an important question (Brown 2015: 5). However, public policy studies have examined expertise as providing evidence for politics, exploring how scientific findings can be accepted as meaningful for policy making (Cairney 2016, 2019; Parkhurst 2017; Bogenschneider and Corbett 2021). In this sense, the effectiveness and precise application of scientific knowledge in politics tend to be taken for granted. This has led to investigation into a series of specific empirical studies on scientific advice (Brown 2015: 8).

Building on these approaches, this article re-describes issues related to the scientific advice for politics from another perspective, namely, that of social systems theory. Niklas Luhmann’s conception of systems theory provides us with an innovative means of observing modern society as a communications-based and functionally differentiated society. This approach may contribute novel insights for the problems of scientific advice in politics.

As a case study, we consider the COVID-19 measures adopted by the Japanese government following February 2020. The role of scientific advice in Japan’s COVID-19 response has been challenging to understand in terms of the positioning of the advisory body and its politicization. While the domestic discourse has focused on the merits and demerits of this politicization, this article investigates the politicization of the advisory body to contribute to the development of a research program on the relationship between politics and science in times of crisis, developing this case that can clarify the concept of structural coupling in social systems theory.

## Scientific Advice from a Systems Theoretical Perspective

In Luhmann’s social systems theory, society is considered to be autonomous, not a mere aggregate of individuals but a communications system. Modern society is composed of multiple autopoietic functional systems that operate autonomously and in a closed manner. Such functional systems are neither dependent on nor regulated by the operations of others, instead creating their own operations based on their own criteria (codes and programs etc.) while taking other systems as their own environment.

A challenge in the theory of a functionally differentiated society containing self-referential systems is how the insights of intersystem relations can be deepened. According to Luhmann, functional systems cannot be grasped in the form of decomposition schemas based on groups, organizations, or nation-states, but are actually emergent processes, in which political, economic, legal, religious, and scientific systems are formed through communication (Schimank 2005). These characteristics of functional systems are oriented through the communication process as their basis (Luhmann 1997). In other words, the functional differentiation of society is a historical phenomenon, established through the accumulation of communicative boundary maintenance over time (Luhmann 1980; Sakai 2021; Steiner-Khamsi 2021: 808).

Thus, the concept of structural coupling describes and explains how intersystem relationships are created (Luhmann 1997: 100ff). Social systems, including functional ones, operate separately but create relationships with their environment. They receive external stimuli from their environment that is formed within a system and then are considered as triggers for their further operation. Functional systems do not directly interact but maintain the autonomy of their operations by observing the environment. Structural coupling indicates this specific construction of the connection between systems and the environment. Thus, only a few aspects of the environment can be grasped by structural coupling (Luhmann 1997: 107).

Luhmann presents structural coupling as follows:

Structural couplings, on the other hand, occur when a system permanently presupposes certain characteristics of its environment and structurally relies on them. (Luhmann 1993: 441, translated by the author)

Regarding structural coupling between functional systems, these “certain characteristics of the environment” are those of certain organizations and institutions (Luhmann 1997: ff1070). Each organization or institution (*Einstellungen*) “structurally relies on certain characteristics of the environment” for each of the multiple functional systems. Luhmann lists the following institutional forms that allow for structural coupling between functional systems (Table).

**Table:** Various forms of structural coupling

politics—economics	taxes and duties
law—politics	constitution
law—economics	property rights/contracts
science—education	university
politics—science	expert advice
education—economics	proof of grades and issuance of transcripts
medicine—economics	medical condition record
art—economics	art commerce (gallery)

Luhmann emphasizes that none of these apparatuses/institutions have a privileged function in society but are rather highly outstanding in a functionally equivalent way (Luhmann 1997: 1077).

Logically supporting this insight is the observation that the only type of social system with the potential to communicate with systems existing in the environment is the organization (Luhmann 1997: 1129; Morten 2007). In *Organization and Decision*, Luhmann describes the relationship between organizations and functional systems as follows:

And that is precisely why society must go beyond functional differentiation and use another principle of system formation to provide itself with ultra-stability and sufficient local capacity to absorb irritations, namely organization. (Luhmann 2000a: 96, translated by author)

An autonomous functional system, as it is maintained by relationships that are constructed with its environment through the organization, is made up of various forms of communication that are not represented by any nation-state, organization, or membership group. The various organizations can process external stimuli locally. It is through such organizations that structural coupling is possible.

Another important aspect of intersystem relations is the complementary nature of structural coupling through operational coupling. Functional systems relate to each other through on the above-mentioned organizations and institutions and direct their own operations separately. However, in some cases, the two systems are coupled not only on that basis, but also through their operations (Luhmann 1993: 440–1). For example, a payment can also signify the fulfillment of a legal obligation; the promulgation of a law can represent political agreement or disagreement (Luhmann 1993: 578); and a doctor’s written confirmation of a medical condition can also be taken as information the patient can give to his/her employer (Luhmann 1997: 788). Where a single communication can operate in multiple functional systems simultaneously and independently and in separate ways, it exhibits “operational coupling.” Operational couplings, it should be noted, cannot replace structural couplings but presuppose them. However, they condense and update mutual irritations and thus allow faster and better coordinated acquisition of information in the systems involved (Luhmann 1997: 788).

## Advice as a Form of Structural Coupling

Following from these theoretical findings, it is reasonable to view scientific advice on policy making during a crisis as an empirical case of structural coupling between political and scientific systems (Luhmann 2000b: 393ff). To understand structural coupling, it is necessary to begin with multiple autonomous systems. Instead of science producing knowledge that is then applied to the political system, scientists, teams of advisors, and clients create different interaction systems (Luhmann 2000b: 394). In place of a linear transfer of knowledge that moves from science to politics, uncertainty becomes the object of analysis (Grundmann and Stehr 2012). The system-system nexus on which advice is premised is not a linkage but only as a reciprocal stimulus through organization, where the question of control in the political system, in which decisions are made by comparing other options, becomes an issue problem for the scientific system (Luhmann 2000b: 395). However, advice, as a structural coupling, should not always be regarded as orderly with respect to a given system’s boundaries, but also as being actually constructed on a case-by-case basis through operations as individual communications within the organization (Sakai 2022). The concept of operational coupling implies the emergent formation of systemic relationships at the level of communication, and structural coupling is the institutional basis for the relationships that are supported by it.

This is exemplified below with the Novel Coronavirus Expert Meeting, a scientific advisory body that was created as part of Japan’s response to the outbreak of the COVID-19 pandemic. The following items are key to a sufficient description of this structural/operational coupling. First, how did the organization exhibit a structural coupling of science and politics and what difficulties did it encounter? Second, how did the organization transform itself in the face of criticism with respect to its status. The issues in dispute in the group and among its critics were the politicization of the experts, characterized as overstepping, and front-loading (Makihara 2020).

Studies of expert panels in Japan’s COVID-19 response have hitherto tended to assume a distinction between the groups of expert, government, and citizen actors. Taking this as a premise, investigations of the politicization of expert meeting have considered it both positively and negatively (Adachi and Sugitani 2020; Hirono 2020; Makihara 2020; Sadamatsu 2021). This approach, however, equates actors’ attributes with institutional boundaries and may fail to describe the specific communicative operations of the expert meeting, which is tasked with the structural coupling of politics and science to deter an infectious disease pandemic. In the expert meeting, experts’ words and actions of experts were not received solely as scientific communications, and the words and actions of government officials were not received solely only political communications. Rather, simultaneous attribution of communication to functional systems occurred. The behavior of the expert meeting as a scientific advisory body during the crisis must be understood more carefully in relation to the perspective of communication systems theory, which considers each event in this context as an operational coupling of politics and science. Further, expert advice as a form of structural coupling does not provide authority but uncertainty (Luhmann 1997: 786). Here, by focusing on the status and role of the organization, we analyze the modalities of the coupling at both a structural and operational level.

## Case Study

The period of analysis for this case study runs from February to June 2020, between the establishment and reorganization of the Expert Panel for the Corona Response in Japan. I examine how the scientific advisory body was established and how its position changed (3.1), how policy makers reacted to the advice of the expert meeting (3.2–3.3), and how the expert meeting itself reflected on its relationship with politics and repositioned itself as a result (3.4–3.5). Sources to be analyzed include third-party verification reports, reportage based on interviews, articles on the Web, and primary documents by the expert meeting.

The members of the panel described their actions as “crossing the Rubicon River as scientists.” That is, they considered themselves has having made suggestions and taken actions that could have a significant impact on policy decisions. The characteristics of the Japanese response to the COVID-19 pandemic have been identified by the expert meeting and government as having a unique character, the “Japan model” (API 2020: 33 − 51). This model developed from the scientific judgment of the members of the expert meeting, cluster tracking, early diagnosis of patients, and assured of health care delivery, as well as behavioral changes seen among citizens, in place of lockdowns or seeking group immunity. However, the Prime Minister’s Office and the Ministry of Health, Labour and Welfare (MHLW) disagreed about how to implement the Japan model.^1^

### Changing Roles of Advisory Organizations

The first expert advisory organization convened in response to the novel coronavirus infection was established in early February 2020 after a passenger of the cruise ship *Diamond Princess*, which had left the port of Yokohama, was confirmed to have contracted the disease in late January 2020. The Novel Coronavirus Infection Countermeasures Advisory Board was established by the MHLW, and its membership was mainly drawn from the members of the expert meeting that had been in charge of the response to the H1N1 influenza pandemic in 2009. At this stage, the board was providing advice in the form of opinions in response to matters discussed by the MHLW’s Headquarters for the Promotion of Countermeasures (API 2020: 114ff).

With regard to growing concerns over the spread of infection in the general population off of cruise ships, The Novel Coronavirus Expert Meeting was established on February 14 as part of the government’s task force, where the Advisory Board was also transferred. Although the meeting lacked a clear legal basis, it was joined by members of the Advisory Board and several other experts. Although it was established within the Cabinet Secretariat, both the location of the meetings and the office associated with it were set up by the MHLW, which created some confusion about where the organization should be understood to belong. Some believe that this confusion was a complicating factor in the relationship between the government and the panel’s experts (Kawai 2021: 21). At this point, although informal meetings (called “study groups”) were being held by members of the meeting (Kawai 2021: 30–31), and the scientific communication among the experts continued to operate, the constituent members played a generally passive role, generally responding to proposals presented by the government. This network later formed the basis for a group that acted independently of government organizations.

As multiple expert advisory organizations were being created, the expert meeting under the government’s task force not only responded to the proposals presented by the government but also recognized the need for its expert side to create its own analysis of the pandemic situation. As a result of this, without the government’s policy input, the expert meeting presented its “Opinions on the Specifics of the Basic Policies for Countermeasures against Novel Coronavirus Infections,”^2^ as well as compiling a proposal for measures to be taken to prevent COVID-19 infection, presenting this to the government. This move was taken because the panel felt that the government’s communications were not well founded in medical evidence and could not be expected to properly instruct the public, which was not properly aware of the risks (API 2020:122ff).

However, there was a backlash from MHLW officials against this dissemination of information by the expert meeting. These officials also made numerous requests in terms of content and format, presented after revisions had already been made, and these officials demanded proceduralist rigor, insisting that future communications documents be approved by going through the ministerial hierarchy. After this date, the expert meeting became more than an advisory body to the government (Kawai 2021: 39). Instead of responding to the government’s inquiries based on its members’ expertise, the expert meeting was now being used to make recommendations to civil society at large, going beyond the government’s strict requirements. This step was taken because the expert meeting was aware of the need for experts to analyze the state of the pandemic, compile a proposal for infection prevention measures, and then present it to the government. Shigeru Omi, the vice chair of the expert meeting, described the issuance of this opinion as the moment when the “first Rubicon River” was crossed (Ogawa and Hashimoto 2020).

### Autonomous Operation of the System: Ad Hoc Advice Usage

Policy makers were ambivalent in response to the “front-loading” opinion by the expert meeting. Although the findings were considered important for policy making, the government implemented measures without consulting the expert meeting, including the requirement to close all schools at once (February 27, 2020) (API 2020.2.27). Some consider that this was because the Prime Minister’s Office felt a sense of crisis after the issuance of the expert meeting’s opinion, and it sought to take the lead in policy making by moving ahead alone, without coordination with the expert meeting (API 2020:130). For example, Shigeru Omi said, “There must be a desire of course to be politicians and take the leadership themselves” (Kawai 2021: 51). The arbitrary policy decisions made by the government without scientific evidence extended to the distribution of cloth masks and economic measures.

Significant discord was seen in the expert meeting, not only in response to the Prime Minister’s Office’s actions but also with the bureaucratic organization as a whole. After the expert meeting issued its opinions on March 3 to the public, a series of negotiations took place with the MHLW and local governments even over details of the wording of each document; this repeated tendency toward coordination and intervention stemmed from a so-called principle of infallibility in the bureaucracy. According to this principle, official documents should be created on the assumption that the government never makes mistakes. The stance of the expert meeting, which sought to disseminate the most accurate and extensive information available at the time, is in conflict with this, and due to adjustments required by government officials, the public explanations sometimes fell short. The MHLW sought to include in its document some content that had not been discussed by the expert meeting, and even requests made by the expert meeting of the MHLW were meticulously checked and revised by the MHLW in advance, as the MHLW did not want to examine what could not be printed(Kawai 2021: 53–8). This close relationship with government officials helped establish the image of the expert meeting for civil society, and, around March 2020, the meeting became recognized as the authority for the government’s measures.

### Interface Between Politics and Science

As noted, the expert meeting functioned as a scientific advisory organization for policy making in the government and bureaucratic organizations. In this context, the technical officers in the MHLW who acted as the interface between the scientists in the meeting and the politicians and officials in the Prime Minister’s Office played a significant role (Kawai 2021: 120). The technical officers selected the members of the first advisory Board in early Februaryand were responsible for the administration and ongoing support of the expert meeting (API 2020: 298; Kawai 2021: 15–6). They themselves were bureaucrats who had expertise in public health and other fields. They were also required to write reports to summarize and publish discussions at the expert meetings while also identifying human resources in municipal health centers, sometimes rejecting measures and policies proposed by experts based on this information. They sought to develop feasible proposals, combining their own scientific knowledge with that of the experts and drawing on the Ministry’s internal practices on the frontlines as well. These technical officers acted as an interface between the experts and the officials.

### Politicization of the Expert Meeting

A major development in the response to the novel coronavirus was the first declaration of a national state of emergency and its subsequent lifting (April 7, 2020, to May 25, 2020). In the Japan model, this declaration was not a lockdown. Instead, it was intended to restrict flows of people and avoiding crowding. After the emergency declaration was issued, informal discussions were held between several members of the expert meeting and the cluster task force and Yasutoshi Nishimura, the minister in charge of COVID-19 countermeasures (Kawai 2021: 125). This discussion allowed them to present the declaration of a state of emergency on a message that was grounded in scientific findings that were based on mathematical models. This enabled the experts to be more directly involved in policy making. According to Omi, the experts’ recommendations in issuing the emergency declaration were “the second Rubicon River”:

We can only make recommendations. It is the government that implements [the policies]. If the government machine does not work, we cannot do anything. The reason we are doing this is because we want to bring down infection rates. (Kawai 2021: 128)

Here, the experts appear to recognize the state of the case from a professional standpoint and present a range of possible policies to policy makers, while also providing a stronger report discussing the government’s actions to control the infection. Minister Nishimura, in response, was overwhelmed by the positive attitude of the expert meeting (Kawai 2021: 124).

By contrast, the expert meeting had very little influence on the subject of lifting of the emergency declaration. The initial decision to declare a state of emergency was made by the Advisory Committee on Basic Response Policies, where, despite the phased schedule for lifting of the emergency declaration, the expert meeting did not make any recommendations, and its discussion of the criteria for lifting the declaration remained vague (API 2020:161), as the committee consisted of all of the members of the expert meeting and several medical professionals from the parent Council of Experts on H1N1 Influenza Countermeasures. In other words, in this phase, the “front-loading” attitude of the experts was overshadowed, and the Prime Minister’s Office took the political initiative for determining to cancel the emergency declaration. The Prime Minister’s Office held that “the expert meeting is useful in tightening the restrictions, but it cannot be responsible for loosening them” and that “if we had followed the experts’ opinions, we would never be able to lift the restrictions” (API 2020:162).

From this point on, however, the expert meeting and its members began to develop a closer relationship with policy makers, mainly those in the Prime Minister’s Office. The press conference that was conducted on the declaration of the state of emergency was held in the presence of then Prime Minister Shinzo Abe, with Omi as the representative of the experts. Omi directly answered questions from the press regarding his areas of expertise and point of view of the expert meeting. Here, the expert meeting was trusted as an advisory body and as one in which politicians (Abe) embraced the experts (Omi) (Kawai 2021: 149). This exhibited a tendency to place the responsibility on the experts and to throw the blame on them.

### Reflections of the Expert Meeting

Following the above-described politicization of the expert meeting, an organizational reflection on this “forward-looking” attitude took place. This resulted in a reorganization of the body (Kawai 2021: 166–169; API 2020:162).

The background to this discussion was the criticism, threats, and lawsuits originating in the public against the expert meeting’s own communications and recommendations. Expressing concern regarding the government’s inability to present substantive recommendations to the public, the expert meeting sought to present information directly to the public. However, members of the expert meeting recognized that they were beginning to show a “front-loading” tendency, so a correction of this politicization was proposed. Specifically, an essay by a public policy researcher who was not a member of the meeting (Makihara 2020) proposed that the meeting be dissolved to ensure that its experts retained their independence. This proposal noted that because the meeting without a legal basis, was unstable and dangerous, it would be necessary to clarify where responsibility lies and improve transparency. It was also feared that the experts could act as a shield for the Prime Minister’s Office. Therefore, it was suggested in the meeting that, as the meeting had not been convened on a legal basis, this grounding be provided and that several subcommittees of experts in various fields be established under the meeting to discuss related issues.

On June 24, the expert meeting released a proposal entitled “For a New Expert Advisory Organization to Prepare for the Next Wave” (Members of the Novel Coronavirus Expert Meeting 2020), and three members of the meeting held a press conference. The panel called this proposal a “graduation thesis”; in it, they identified two problems that they had encountered that went to the question of what form an expert advisory organization should take.

The first concerned the relationship of such a body with the government. The proposal presents the following principle: the expert meeting provides advice from a medical standpoint, and the government makes policy decisions based on its recommendations, but the boundary between the two ceased to be clear from the outside. When the experts disseminated information, the impression was given that the expert meeting had power to decide policy. It was feared that the members of the meeting may have overreached. The second problem concerned the dissemination of information to citizens: the expert meeting’s opinion of February 24 (4.1), in which the expert meeting recognized that it had “created both expectations and doubts about the expert meeting beyond its original role,” was a direct call to action by citizens, without government involvement, based on a sense of crisis at the expert meeting. From this, the public may have come to see no boundary between the government and the scientific community. From these reflections, the expert meeting envisioned a new expert advisory body that would be set up, for which the scope of responsibility and roles of advisory organizations would be established in advance. Additionally, the expert meeting made an account of risk communication and anticipated issues through the promotion of infectious disease control, including ethical, legal, and social issues.

This press conference met with some opposition from the MHLW. A representative of the expert meeting regarded its operations as “forward-looking,” but the MHLW considered that “joint effort” between the experts and the government took place at the meeting (Kawai 2021: 188). For the government side, the fact that the expert members would go beyond giving “advice” to making specific recommendations was factored in. The experts’ perception that they had sent their messages across the “Rubicon River” was very different from the thinking of policy makers.

Following this, the existing expert meeting was reorganized as an advisory body, now with clear, open, and legal grounding, under which subcommittees in several fields were established to integrate opinions. The government then coordinated policy. The expert meetings were replaced by Subcommittee on Countermeasures for Infectious Diseases of New Coronaviruses. However, even after this, experts in advisory organizations faced further “Rubicon Rivers” over economic measures and the form of the Olympic Games. As a result, the Subcommittee rather strengthened its ties with policy makers.

## System Theoretical Re-description

The first aspect demonstrated by the case studies in this article is the autopoietic closure of the political system. The pandemic originated from the cruise ship stimulated the political and scientific system, as information from the environment conditioned the further functioning of both systems. In this context, the expert meeting was initially set up as an internal organization of the political system, because it was organized by technical bureaucrats with experience in solving public health problems in the past. In fact, scientific knowledge was frequently used only to contribute to government decisions. This scenario is an empirical demonstration that scientific knowledge is only information belonging to the environment for the political system and that it is the stimulus for self-referential operational connections that lead to decisions. Functional differentiation can also be a fundamental social structure in crisis communication. Predicting that such functional differentiation between politics and science will take different operational forms in each case is easy. However, if a situation exists in which scientifically idealistic findings are directly implemented in politics, irrespective of political systemic rationality, then it could be a sign of a de-differentiated society.

Second, the expert meeting can be theoretically described as an organization that is simultaneously positioned inside and outside the scientific system. Through the form of *advice*, this organization was able to produce not only communication oriented to contribute to the operation of the political system but also the simultaneous production of communication based on reference to the scientific system, which could be at conflict with the political. In particular, the expert meeting functioned as an organization that provided independent scientific validation from the perspective of infectious diseases and public health, while serving as one that contributed to the operation of the scientific system. Furthermore, expert meetings gradually gained organizational autonomy and began to produce communication that could be connected to the operations of the political and scientific systems, organizing the informal research group at the same time. This organizational formation enabled the expert meeting to become the core of the structural coupling of the political and scientific systems and to establish itself as an organization that produced an operative coupling at the communication level. Moreover, we can also theoretically understand that the expert meeting as an organization cannot represent the scientific system. Certainly, expert meetings produced scientific communication that exceeded practical knowledge to contribute to politics but were also observed as an environment for the scientific system. This notion is evident in the critical statements made by many experts on infectious diseases and public health about the scientific recommendations and analytical procedures of expert meetings (e.g., Okada 2020).

The advantage of autopoietic social systems theory is that it considers the components of the system not as actors but as communication to which meaning is attributed to each operation. Third, based on this theoretical tool, actor-based analysis is clearly insufficient for understanding the attribution of communication to politics and science in expert meetings and, thus, to the intersection between political and scientific communication. On the one hand, a few members of government organizations, including technical bureaucrats, possessed scientific expertise and heeded the recommendations of expert meetings without being merely politically interested. On the other hand, scientists in expert meetings did not ignore the feasibility of proposals but attempted to make recommendations based on policy rationale while taking scientific validity into consideration as much as possible. In other words, the expert meeting was not a site of conflict between the political and scientific systems. Instead, it was an interface at which communicative rearrangements constantly occurred at the operation level, which could not be reduced to either of two systems This idea would correspond to the conversation sphere between systems as conceptualized by Hutter (1989) in an extension of the structural and operational coupling concept purported by Luhmann.

Finally, how can the self-reflexive reorganization of expert meetings, as discussed in Sect. 3.5, be theoretically redescribed? This reorganization was undertaken to redefine its legal and social position; as a result, the expert meeting as a subcommittee became an intellectual resource that political decision makers could utilize to a greater extent than they previously did. However, this notion does not mean that expert meetings were completely included into the political system. Instead, the political system was able to externalize the basis for its decisions by recognizing that its environment, that is, the scientific system, could be observed through this organization, because the subcommittee reflected on “what kind of organization it is”. In this sense, the reorganization even strengthened the structural coupling. Through the form of advice, the structural coupling of the political and scientific systems, thus, occurred in a situation in which political and scientific communication are sharply differentiated in practice but can be synchronized through organization.

## Further Discussion

The semantic and organizational transformation of the expert meeting as a part of Japan’s COVID-19 response is an example of the structural/operational coupling of the political system and the scientific system mediated through organizations, manifested locally under the form of advice. This theoretical redescription raises additional theoretical and empirical challenges, notably with regard to structural and operational couplings, the exploration of selection mechanisms, and the limitations of each functional system. First comes the question of how scientific knowledge is selected in advisory organizations. This involves the issue of membership or the inclusion/exclusion principle of organizational systems ((Luhmann 2000a). In the Japanese case, the bureaucracy is at the core of the principle of selecting organizational members of advisory boards of experts. The second question, an understanding that requires very detailed empirical research, regards how the recommendations of the advisory organizations are coordinated with policy makers. Thus, it should be asked whether the operation of the scientific system can be regarded as a stimulus for the understanding of problems specific to the political system (e.g., interest groups, medical associations, etc.) and as a resource for their solution. A comparative analysis of multiple cases is needed to determine what defines both points.

Most scholars agree that a relationship of trust is needed between policy makers and scientist in times of crisis (API 2020:279; Cairney and Wellstead 2021). However, when this relationship of trust is established, this reflects the trust of the systems themselves in the mechanisms that relate to functional systems, such as structural and operational couplings.

## Data Availability

Not applicable.

## Footnotes

1 For example, the Prime Minister’s Office sought to expand PCR testing, but the Ministry of Health, Labour and Welfare halted this initiative. There were also administrative inspections that sought to monopolize the right to PCR testing, and fears of overwhelming the medical care system were among the points of concern.

2 Despite apparent internal conflicts, the expert meeting disseminated information at the suggestion of a member of the meeting who specialized in social medicine (Kawai 2021: 25).
